# Quantifying gene expression: the importance of being subtle

**DOI:** 10.15252/msb.20167325

**Published:** 2016-10-26

**Authors:** Gustavo Monteiro Silva, Christine Vogel

**Affiliations:** ^1^ Department of BiologyCenter for Genomics and Systems BiologyNew York UniversityNew YorkNYUSA

**Keywords:** Genome-Scale & Integrative Biology, Post-translational Modifications, Proteolysis & Proteomics, Transcription

## Abstract

Gene expression is regulated at both the mRNA and protein level through on‐off switches and fine‐tuned control. In their recent study, Edfors *et al* (2016) use highly accurate, targeted proteomics methods and examine to what extent the amount of protein produced per mRNA transcript varies across different tissues. They find that the bulk part of protein concentrations is set at a per‐gene level: This relationship, the protein/mRNA ratio, is constant across cell types and tissues, but varies by several orders of magnitude across genes.

In recent years, a flurry of studies has examined the relationship between mRNA and protein concentrations across genes, with sometimes contradicting findings (Liu *et al*, 2016). In yeast, protein concentration can be predicted very well from mRNA concentration (Csardi *et al*, 2015). On the other hand, in mammalian cells the correlation has been shown to be much lower and variable depending on the cell type and state. The situation becomes even more complicated for cells that have been subjected to a stimulus. For example, during the response of dendritic cells to lipopolysaccharide treatment, each protein's concentration appears to be largely determined by its mRNA concentration, as expected from a condition known to involve extensive transcriptional regulation (Jovanovic *et al*, 2015). In contrast, in mammalian cells subjected to protein misfolding stress, the correlation between protein and mRNA concentrations breaks down and extensive regulation occurs at both the mRNA and protein level (Cheng *et al*, 2016).

The lack of correlation between mRNA and protein concentrations has often been attributed, at least partly, to the high measurement noise of proteomics methods. The inherent variability of approaches such as shotgun proteomics that sample a subset of the proteome has often been counteracted by careful statistical evaluation of the data and the use of error models that estimate and “subtract” measurement noise. However, more recent proteomics methods that measure fewer proteins with high accuracy (instead of many proteins with low accuracy) allow producing highly reliable quantitative measurements.

Edfors *et al* (2016) used one of these methods, PRM (parallel reaction monitoring), to measure the concentrations of 55 proteins in 20 different human cell lines and tissues at high resolution and low error rate (Edfors *et al*, 2016). In addition, they used histone abundances to robustly normalize for the total number of cells in the sample. This approach produced a unique gold standard of protein concentration data that, albeit small, can be used to answer many fundamental biological questions. One of them goes back to the issue discussed above: What is the correlation between mRNA and protein concentrations across the analyzed genes? To answer this question, Edfors *et al* (2016) compared RNA‐seq‐based estimates of mRNA concentrations to the protein measurements and found a reasonable correlation between the two entities across genes (average Pearson's *r* of ~0.60 at a log–log scale) consistent with earlier findings using shotgun approaches (Schwanhausser *et al*, 2011; Wilhelm *et al*, 2014).

Notably, the authors then moved on to the next important question: How much does this protein–mRNA relationship for a given gene *change* across different tissues and cell lines? Is it hardwired into the genome or does tissue‐specific regulation at the protein level change the protein/mRNA ratio? To answer this question, the authors compared protein and mRNA measurements across tissues for one gene at a time (Edfors *et al*, 2016). Importantly, they focused only on mRNAs that were present across all tissues and excluded the genes for which the absence/presence of mRNA contributes to tissue‐specific expression regulation. They found that the protein/mRNA ratio is largely conserved across tissues for a given gene but varies widely across different genes. This indicates that while the mRNA concentration might vary, the amount of translation (and degradation) of the gene's transcript appears to be a property of the gene itself, is encoded in its sequence, and does not change.

When taking into account the gene‐specific protein/mRNA ratios, the correlation between mRNA and protein concentrations within a tissue and across genes vastly increases, up to a median *r* of ~0.93 at a log–log scale. More than 85% of a gene's expression variation is determined by variation at the mRNA level, once the gene‐specific translation/protein degradation rate is considered—a number very similar to what has been found for yeast (Csardi *et al*, 2015).

Does this mean that protein concentrations are entirely set by mRNA concentrations and therefore there is no need to go through the much more demanding proteomics route to measure protein concentrations in different cells? Not quite so. On the one hand, indeed the bulk part of a protein's concentration can be well predicted by its mRNA concentration, as Edfors *et al* (2016) show.

On the other hand, while across genes the range of concentrations spans several orders of magnitude, we need to remember that biology is in many cases much more subtle than differences this large. In many known examples, fold changes across conditions as small as twofold or even lower have substantial functional consequences, which is one reason why a twofold up‐ or down‐regulation of a gene is often used as a cutoff in differential gene expression analysis. When examining the data by Edfors *et al* (2016) more closely, we observe remaining unexplained variance that might account for some of the differences between the cell and tissue types. For example, as shown in Fig 1, at similar mRNA concentrations, CD81 protein concentration can vary by approximately two orders of magnitude across different tissues. In comparison, MEF2D mRNA concentrations can vary by up to tenfold at similar protein concentrations.

**Figure 1:**
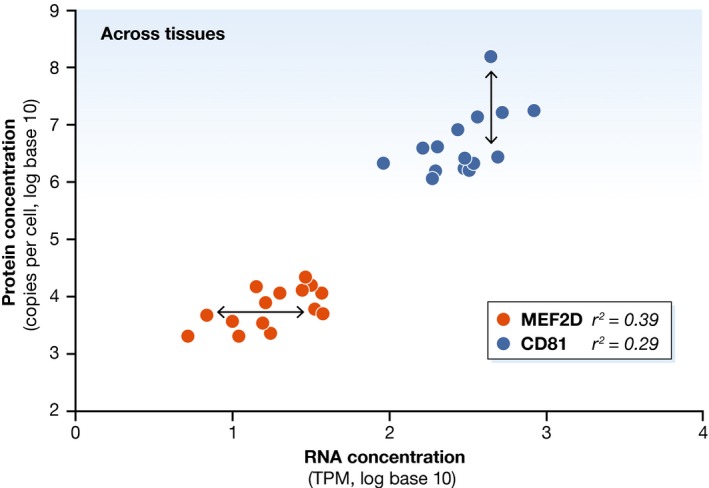
Subtle changes characterize different tissues The graph shows the mRNA and protein concentrations for two example genes, MEF2D and CD81, across several tissues and cell lines (log base 10; TPM, transcripts per million). The concentrations are well correlated at a log–log scale. Some variation remains (double arrows): At similar protein concentration, MEF2D's mRNA concentration varies across tissues up to eightfold, while, vice versa, CD81's protein concentration varies over close to two orders of magnitude (100‐fold) for a given RNA concentration.

Therefore, we need to continue paying attention to details such as small changes in protein expression, because they contribute to defining tissue‐ and cell‐type‐specific functions. Undoubtedly, turning a gene's transcription on or off is a major contributor to tissue specificity and gene expression response. However, on top of this switchlike regulation, protein‐level regulation appears to fine‐tune expression levels (Vogel & Marcotte, 2012; Liu *et al*, 2016). Since these changes are rather subtle, further development of experimental and computational methods that enable us to accurately and reproducibly quantify these small differences is essential. Currently available genomewide methods have enabled us to sketch an overall picture of gene expression levels, and now it is time to use the fine brush to add nuanced colors to this picture.
